# A Multivariate prediction model for amlodipine therapeutic efficacy in pediatric primary hypertension

**DOI:** 10.3389/fendo.2025.1542276

**Published:** 2025-02-11

**Authors:** Yao Lin, Hui Wang, Yaqi Li, Yang Liu, Yanyan Liu, Hongwei Zhang, Yanjun Deng, Lin Shi

**Affiliations:** Department of Cardiology, Children’s Hospital, Capital Institute of Pediatrics, Beijing, China

**Keywords:** pediatric primary hypertension, amlodipine, predictors, antihypertensive therapy, therapeutic efficacy

## Abstract

**Background:**

There are currently no biomarker-based prediction models for amlodipine therapeutic efficacy in pediatric hypertension. This study aimed to identify potential biomarkers and establish a biomarker-based model for predicting amlodipine therapeutic efficacy in pediatric primary hypertension (PH).

**Methods:**

From January 2022 to December 2023, 165 children and adolescents with PH prescribed amlodipine were recruited at our department for a prospective observational study. Patients were grouped into Responders and Non-responders after one month treatment. The baseline data in the two groups were analyzed to identify variables associated with amlodipine treatment responsiveness; furthermore, a nomogram prediction model was established based on those potential predictors derived from multivariate regression analysis. This model’s discrimination and calibration were evaluated by a series of statistical methods and internal validation was done using the bootstrap sampling method (1000 resamples).

**Results:**

Eighty-nine patients responded to amlodipine while 76 did not. After statistical adjustment, 4 variables were found to be independently associated with therapeutic efficacy, including hyperinsulinemia (OR = 3.000, 95% CI: 1.409-6.386, *p*  = 0.004), insulin resistance (OR = 2.354, 95% CI: 1.032-5.370, *p* = 0.042), the baseline plasma Endothelin-1 level (OR = 0.627, 95% CI: 0.532-0.740, *p* < 0.001) and amlodipine dosages (OR = 1.743, 95% CI: 1.400-2.169, *p <*0.001). Compared to the baseline model, the full model with the four variables had a good calibration with an area under the curve (AUC) of 0.967 (95% CI: 0.945-0.990), yielding a sensitivity and a specificity of 91.0% and 92.1%, respectively; the clinical decision curve showed a positive net benefit. Additionally, a nomogram model was established based on the four variables and evaluated by bootstrap internal validation with the c-statistic of 0.865 and the calibration curve being close to the ideal line (*p* > 0.05).

**Conclusion:**

A nomogram model with high predictive value for amlodipine therapeutic efficacy in pediatric PH was established. This model may be potentially applied to guide the selection of amlodipine for the treatment of pediatric PH.

## Introduction

Primary hypertension (PH) is now the dominant type of hypertension seen in children and adolescents, affecting approximately 11% of 18-year-olds (1–5). The prevalence of primary hypertension in Chinses children aged 7-17 years increased from 1.4% in 1991 to 2.9% in 2015 as shown by the China Health and Nutrition Survey 1991-2015 (6). A systematic review and modelling study revealed that the prevalence among Chinese children aged 6-18 years was 3.1% in 2020 (7). Effective management of pediatric PH to reduce elevated blood pressure is of critical importance to prevent subclinical target organ damage during childhood and reduce cardiovascular risk in adulthood (2–5). However, there are no individualized medication regimens as initial therapy for pediatric hypertension according to the current American, European and Chinese guidelines (8–10). Calcium channel blockers (CCBs) along with angiotensin-converting enzyme inhibitors and angiotensin receptor blockers are used as the first-line antihypertensive agents (11, 12). Amlodipine is a long-acting CCB; in case of incidental noncompliance that often occurs in the pediatric population, it still provides continuous protection (8–10).

Various methods can be used to test drug efficacy in patients, including randomized controlled trials, real-world studies, observational studies and biomarker studies. Currently, amlodipine is primarily chosen for the treatment of pediatric primary hypertension based on the clinical experience of pediatricians (13), and a randomized controlled trial found that the BP control rate of amlodipine in pediatric hypertension was 34.6% (14). In view of these, in order to improve the application of amlodipine as the initial medication for pediatric PH, identification of novel biomarkers that can predict the therapeutic efficacy of amlodipine is needed. Given that endothelin-1 (ET-1), a potent vasoconstrictor, is implicated in the pathogenesis of hypertension (15), and that ET-1 has been shown to interact with calcium channels (16), we hypothesized that changes in blood ET-1 levels reflect the therapeutic efficacy of amlodipine. Therefore, this study was conducted to explore ET-1 and other potential significant predictors and establish a prediction model for amlodipine therapeutic efficacy.

## Methods

### Ethical approval and consent to participate

This study was approved by the Capital Institute of Pediatrics Ethics Committee, Beijing, China (No: SHERLL2022017), in compliance with the principles of the Declaration of Helsinki. Written informed consent was obtained from all study subjects or guardians.

### Study design and subjects

**Figure 1:**
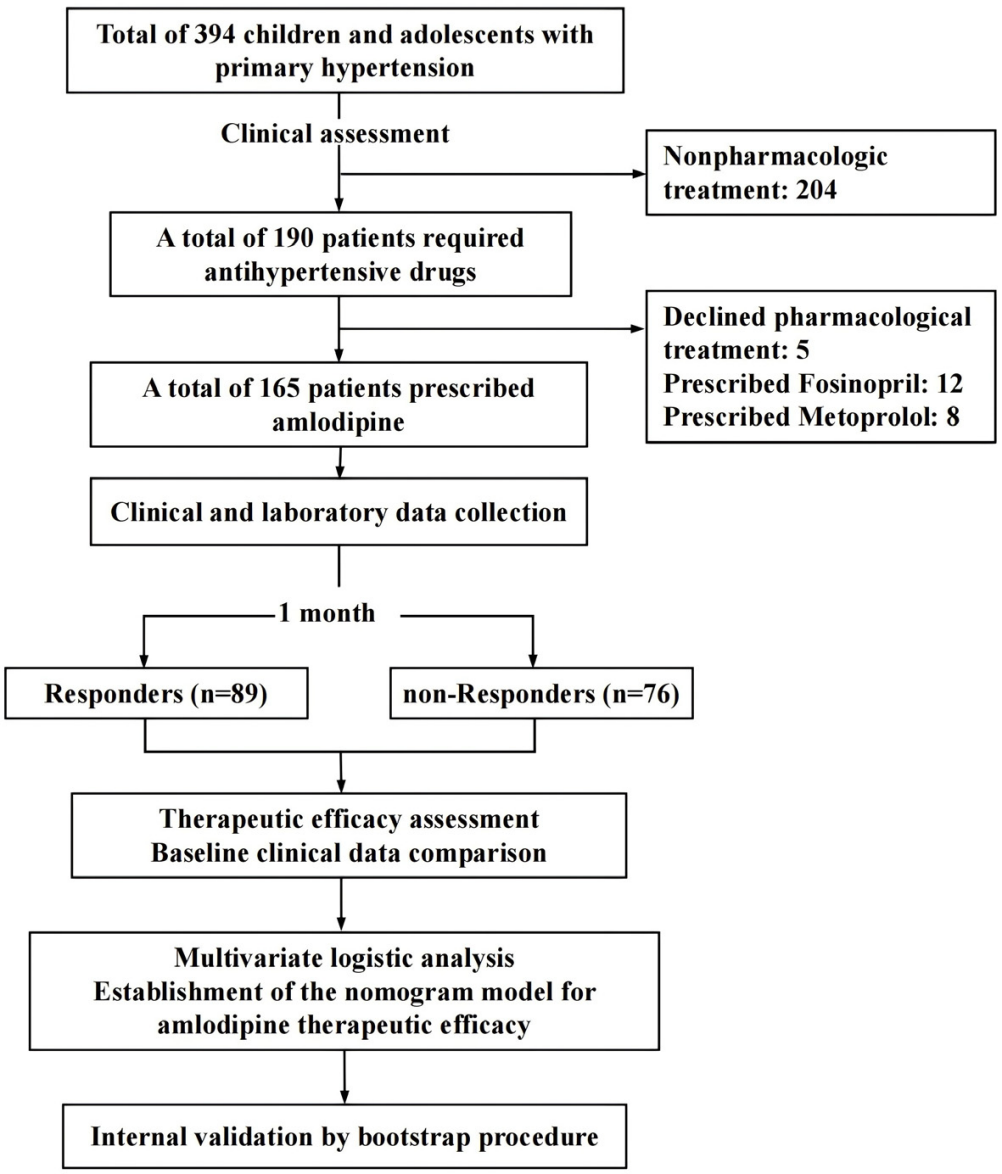
Flowchart of the study. ET-1, endothelin-1.

From January 2022 to December 2023, following the inclusion and exclusion criteria as described below, a total of 165 patients aged 9-17 years with PH who required antihypertensive pharmacological treatment and were prescribed amlodipine were recruited from the Children’s Hospital, Capital Institute of Pediatrics, Beijing, China. The study flowchart is shown in **Figure 1**.

Diagnosis criteria in this study adhered to the “2018 Chinese Guidelines for Prevention and Treatment of Hypertension” (8). Hypertension was diagnosed when systolic blood pressure (SBP) and/or diastolic blood pressure (DBP) ≥ 95^th^ percentile for gender, age, and height on ≥ 3 separate occasions; hypertension stage 1 was defined as SBP and/or DBP ranging from 95^th^ percentile to 99^th^ percentile + 5 mmHg; and hypertension stage 2 was defined as SBP and/or DBP ≥ 99^th^ percentile + 5 mmHg.

Inclusion criteria were as follows: the patients were in need of antihypertensive medication with at least one of the following indications: 1) symptomatic hypertension; 2) stage 2 hypertension; 3) hypertensive target organ damages; and 4) stage 1 hypertension without response to 6-month lifestyle intervention (8). Secondary hypertension was ruled out in all participants using tests selected based on clinical presentation. Major tests included thyroid function assays, plasma renin and aldosterone measurement, and blood creatinine measurement and urinalysis. Patients with white coat hypertension was excluded by ambulatory blood pressure monitoring.

### Clinical data collection and blood pressure measurement

We collected demographic and anthropometric data of all subjects, including gender, age, height, weight, and body mass index (BMI). Blood pressures (BP) at baseline and post-treatment were recorded. The blood pressure was measured using the auscultation method (8). Laboratory data before treatment were also collected for the multivariate analysis, including fasting serum glucose, insulin, C peptide, triglyceride, cholesterol, creatinine, estimated glomerular filtration rate (eGFR), serum calcium, plasma ET-1, 24 h urinary calcium, urinary microalbumin/creatinine ratio (UACR), and *CYP3A5* gene polymorphism. Insulin resistance (IR) was identified by homeostatic model assessment (HOMA) index (17). Left ventricular hypertrophy (LVH) was assessed by echocardiography, and the left ventricular mass index (LVMI) and relative left ventricular wall thickness (RWT) were calculated as described in our previous study (18).

### Protocol of treatment and follow up

All participants were prescribed and received an initial 5 mg daily dose of amlodipine. According to a previous study (19), BP was assessed 5 to 7 days and 2 weeks after the initial treatment. If the BP did not achieve the goal, i.e., BP of 95^th^ percentile for gender, age, and height at each visit, the dose increased by 2.5 mg with a maximal daily dose of 10 mg. It has been shown that the BP level is usually stabilized one month after amlodipine administration (14, 20). Accordingly, we evaluated BP reduction one month after amlodipine treatment. If patients took the maximal dose of amlodipine without achieving the goal BP during the study period and had symptoms associated with high blood pressure or risks of new-onset target organ damage, their participation in the study was terminated.

### Definitions of responders and non-responders

The subjects were grouped into Responders and Non-responders according to the first month’s evaluation results. Responders had SBP and DBP < 95th percentile for gender, age, and height according to the “2018 Chinese Guidelines” (8), while Non-responders did not achieve this goal. To determine the sample size needed to ensure sufficient statistical power, a preliminary study was performed, which showed baseline ET-levels of 1.88 ± 0.65 pg/mL and 1.14 ± 0.41 pg/mL in Responders and Non-responders, respectively. With the Type I error probability set at 0.05 and the statistical power at 80%, a minimum sample size of 25 in each group was required as calculated by PASS (Power Analysis and Sample Size) software, version 15 (National CSS, Inc., Wilton, USA).

### Statistical analysis

All statistical analyses were done using the SPSS 23.0 software (IBM Corporation, Armonk, USA) and R coding platform version 4.2.2 for windows (R Foundation for Statistical Computing, Vienna, Austria).

Normality of continuous variables was determined by the Kolmogorov-Smirnov test. Normally distributed data are expressed as mean ± standard deviation and analyzed using the independent *t* test. Non-parametric data are expressed as median (interquartile range) and analyzed using the Mann-Whitney U test. Chi-squared test was applied for the comparison of categorical data between groups. Variables significantly associated with treatment responsiveness were identified by binary logistic regression analysis before and after adjusting for confounding factors. To appraise the indispensable contribution of significant variables identified, both discrimination and calibration statistics were employed, including Akaike information criterion (AIC), Bayesian information criterion (BIC), Hosmer-Lemeshow (HL) test, net reclassification improvement (NRI), integrated discrimination improvement (IDI), and the area under the receiver operating characteristic curve (AUROC). The clinical net benefit by adding significant variables to the basic model was determined by decision curve analysis (DCA). In the DCA, if the predictor line was higher than the reference line, it suggested that the predictor had a positive clinical value. Then, a nomogram model was built based on significant variables, and its predictive performance was assessed by both C-index and calibration curve as described elsewhere (21). Finally, to test the robustness and validity of our findings, internal validation was done by the Bootstrap procedure, simulating 1000 replications.

## Results

### Demographic and baseline characteristics of all subjects

A total of 165 patients with a median age of 13.0 (2.0) years completed the study without withdrawal or cessation of participation. Of all patients, 77.6% were obese and 80.6% were male. The baseline SBP and DBP were 142.0 (13.0) mmHg and 80.0 (13.0) mmHg respectively. Characteristics of all participants are presented in **Table 1**.

**Table 1:** Demographic and baseline characteristics of all subjects and their comparison between responders and non-responders.

Parameters	All subjects (n=165)	Responders (n=89)	Non-responders (n=76)	*p* value
Demographic parameters				
Age (year)	13.0 (2.0)	13.0 (2.0)	13.0 (2.0)	0.349
Gender (M/F)	133/32	76/13	57/19	0.137
BMI (kg/m2)	28.06 ± 4.90	27.78 ± 4.69	28.39 ±5.14	0.426
Obesity/non-obesity	128/37	66/23	62/14	0.341
Stage1/stage2	23/142	16/73	7/69	0.163
Baseline SBP (mmHg)	142.0 (13.0)	141.0 (12.0)	144.0 (13.0)	0.129
Baseline DBP (mmHg)	80.0 (13.0)	80.0 (13.0)	83.0 (13.3)	0.064
Renal function				
Serum creatinine (µmol/L)	56.45 ± 12.01	58.16 ±11.78	54.46 ±12.05	0.048
eGFR (ml/min per 1.73m2)	105.66 (18.96)	103.65 (18.95)	108.5 (22.06)	0.042
UACR	5.07 (4.29)	4.74 (3.20)	6.00 (6.16)	0.004
Glucose metabolism				
Serum glucose (mmol/L)	4.61 ± 0.47	4.56 ± 0.44	4.67 ± 0.50	0.164
Serum insulin (μIU/mL)	20.10 (15.20)	19.10 (11.30)	24.70 (20.39)	0.006
Hyperinsulinemia (%)	62 (37.6)	24 (27.0)	38 (50.0)	0.004
Serum C peptide (ng/mL)	3.32 (1.43)	3.04 (1.25)	3.64 (1.61)	0.019
HOMA index	4.20 ± 3.14	3.65 ± 2.50	5.00 ± 3.66	0.004
IR (%)	118 (71.5)	57 (64.0)	61 (80.3)	0.033
Lipid metabolism				
Serum triglyceride (mmol/L)	1.19 ± 0.79	1.15 ± 0.88	1.24 ± 0.74	0.27
Serum cholesterol (mmol/L)	3.82 ± 0.82	3.75 ± 0.76	3.87 ± 0.89	0.74
Serum and urine calcium				
Serum calcium (mmol/L)	2.43 ± 0.09	2.43 ± 0.10	2.43 ± 0.09	0.934
24h urine calcium (mmol)	1.87 (1.83)	2.21 (1.90)	1.71 (1.60)	0.024
Other blood examinations				
Plasma ET-1 (pg/mL)	1.46 (0.82)	1.84 (0.83)	1.20 (0.53)	<0.001
CYP3A5 genotype GG (%)	82 (49.7)	49 (55.1)	33 (43.4)	0.182
Enchocardiogram				
LVMI (g/m2.7)	28.04 (6.50)	28.04 (7.43)	27.96 (8.12)	0.884
RWT	0.32 (0.05)	0.32 (0.05)	0.33 (0.06)	0.334
LVH (%)	40 (24.2)	20 (22.5)	20 (26.3)	0.695
Amlodipine dosage (%)				
5mg/d	104 (63.0)	71 (79.8)	33 (43.4)	<0.001
7.5mg/d	34 (20.6)	14 (15.7)	20 (26.3)	
10mg/d	27 (16.4)	4 ( 4.5)	23 (30.3)	

BMI, body mass index; SBP, systolic blood pressure; DBP, diastolic blood pressure; eGFR, estimated glomerular filtration rate; UACR, urinary microalbumin/creatinine ratio; HOMA, homeostatic model assessment; IR, insulin resistance; ET-1, endothelin-1; LVMI, left ventricular mass index; RWT, relative left ventricular wall thickness; LVH, left ventricular hypertrophy. The continuous variables were presented as mean ± standard deviation or median (interquartile range).

### Blood pressure reduction after one-month treatment with amlodipine

The participants had SBP and DBP reductions from 142.0 (13.0) to 125.0 (10.0) mmHg and 80.0 (13.0) to 75.0 (10.0) mmHg respectively (*p* < 0.001). Drug dosages increased in 61 patients 5 to 7 days or 2 weeks after the start of treatment. Eight nine patients responded to amlodipine while 76 did not, with a blood pressure control rate of 54.0%. The Responders had a significant SBP and DBP decrease compared to the Non-responders [20.0 (14.0) mmHg vs 12.0 (11.3) mmHg for SBP, *p* < 0.001; 8.0 (11.0) mmHg vs 5.0 (12.3) mmHg for DBP, *p <* 0.01].

### Univariate analysis

The comparison of baseline data between the Responders and Non-responders showed that a total of 11 factors were significantly different between the two groups of patients (**Table 1**).

### Multivariate regression analysis

Based on the results of univariate analysis, multivariate regression analysis was performed and showed that six variables were significantly associated with amlodipine responsiveness, including hyperinsulinemia, IR, serum creatinine concentrations, eGFR, ET-1_b (calculated as 4 × plasma ET-1/SD [standard deviation]) and amlodipine dosages. After adjustment of age, gender and BMI, only four factors, i.e., hyperinsulinemia, IR, ET-1_b and amlodipine dosages remained to be independently associated with amlodipine therapeutic efficacy (**Table 2**).

**Table 2:** Identification of significant factors associated with amlodipine responsiveness by univariate analysis.

Significant factors	Unadjusted			Adjusted		
	OR	95%CI	*p* value	OR	95%CI	*p* value
Serum creatinine	0.974	0.949-1.000	0.05	0.968	0.929-0.008	0.119
eGFR	1.021	1.002-1.040	0.027	1.021	1.000-1.043	0.05
Hyperinsulinemia	2.708	1.415-5.183	0.003	3.000	1.409-6.386	0.004
IR	2.283	1.121-4.652	0.023	2.354	1.032-5.370	0.042
Plasma ET-1_b	0.651	0.556-0.761	<0.001	0.627	0.532-0.740	<0.001
Amlodipine dosage	1.628	1.336-1.984	<0.001	1.743	1.400-2.169	<0.001

eGFR, estimated glomerular filtration rate; IR, insulin resistance; ET-1, endothelin-1; ET-1_b, calculated as 4 × ET-1/SD (Standard deviation).

### Establishment of a nomogram model to predict amlodipine therapeutic efficacy

**Figure 2:**
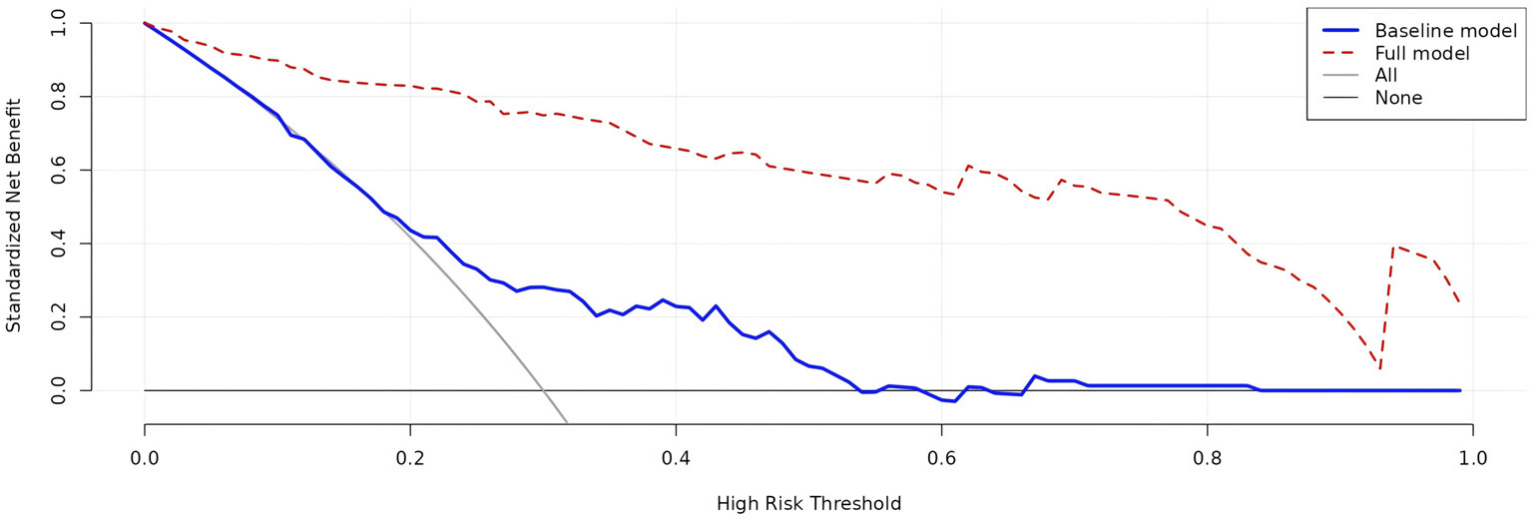
Decision curve analysis of the net benefits related to the efficacy prediction gained by the full model with the four significant variables added to the baseline model. The blue line presents the baseline model, while the red one presents the full model. The baseline model includes age, gender and body mass index.

**Figure 3:**
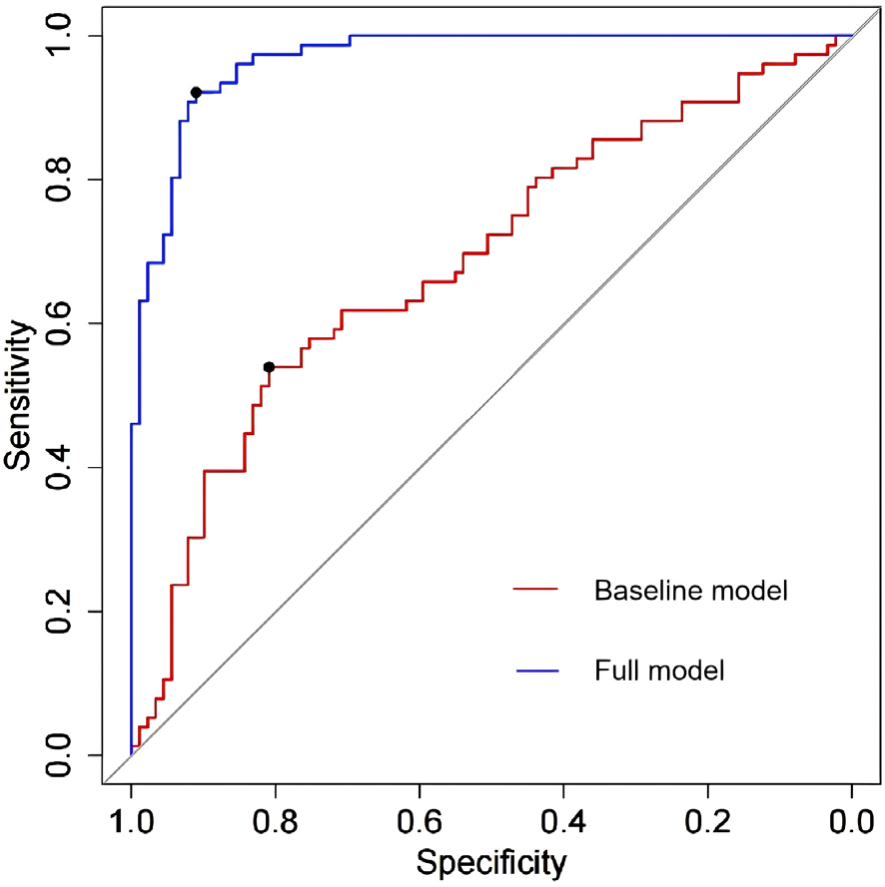
The ROC analysis of the baseline model and full model with the four significant variables. The area under the ROC (AUR) of the baseline model and full model is 0.686 (95% CI: 0.945-0.990) and 0.967 (95% CI: 0.945-0.990) respectively, which means that the four significant variables yield great contribution to the model.

**Figure 4:**
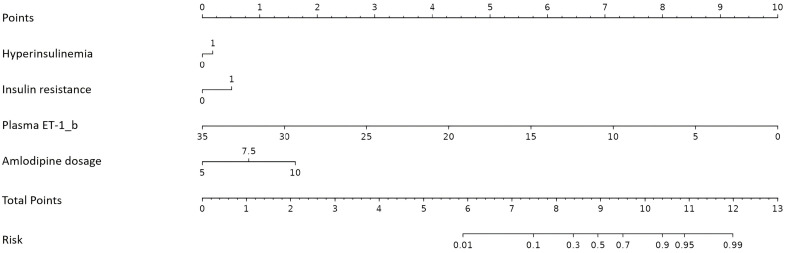
Nomogram for the prediction of amlodipine therapeutic efficacy. ET-1, endothelin-1; ET-1_b is calculated as 4 × plasma ET-1/SD.

The four independent factors were assessed by a series of statistics for their discriminative ability and goodness of fit (**Table 3**) and the net benefits gained by adding the factors to the baseline model that included gender, age and body mass index (**Figure 2**). Compared to the baseline model, ROC analysis showed that the addition of the four significant variables had an AUC=0.967 (95% CI: 0.945-0.990) with the sensitivity and specificity of 91.0% and 92.1%, respectively (**Figure 3**). Furthermore, as shown in **Figure 4**, the nomogram model had a maximum prediction accuracy of 99%. The amlodipine therapeutic efficacy was positively associated with plasma ET-1_b while negatively associated with hyperinsulinemia, IR and amlodipine dosages.

**Table 3:** The predictive performance of the full model with the 4 significant variables added to the baseline model.

Statistics	Baseline model*	Full model
AIC	253	153
BIC	318	271
NRI (*p*)	<0.001	
IDI (*p*)	<0.001	
HL test (*p*)	0.762	0.836
AUCROC	0.686	0.967
AUCROC (*p*)	<0.001	

AIC, Akaike information criterion; BIC, Bayesian information criterion; HL test, Hosmer-Lemeshow test; NRI, net reclassification improvement; IDI, integrated discrimination improvement; AUROC, area under the receiver operating characteristic. *Variables in the baseline model included age, gender and body mass index.

### Evaluation of the nomogram model

**Figure 5:**
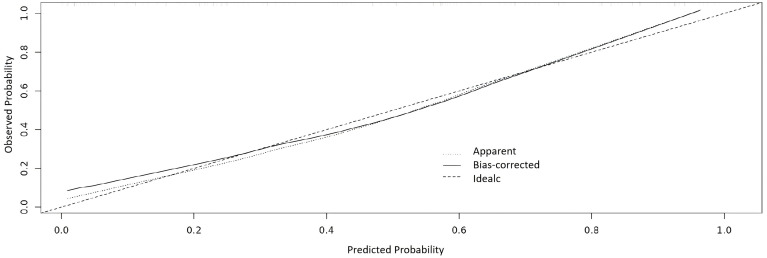
Calibration curve of the nomogram. The X axis is for the predicted probability and Y axis is for the observed probability.

The nomogram showed a good discrimination with a C-index of 0.865, and the calibration curve was close to the ideal line with the mean absolute error of 0.029, indicating a good prediction effect (**Figure 5**).

Internal validation was performed using bootstrapping with 1000 repetitions. The accuracy was 0.761, and the kappa value is 0.517, suggesting the model was stable.

## Discussion

Blood pressure control is of great importance in pediatric PH (2–5). However, there are currently no predictive biomarkers or models for the selection of initial medications. In view of that the BP control by pharmacological treatment in children and adolescents is far from achieving the goal (9, 13), a biomarker-based prediction model that can predict drug efficacy is needed to improve this situation. In this present study, we first identified several parameters substantially different between Responders and Non-responders. The hyperinsulinemia, IR, plasma ET-1 levels and amlodipine dosages were further found to be independently associated with amlodipine therapeutic responsiveness. A nomogram model established based on these factors was shown to have a good discrimination and calibration capacity.

As one of the first-line antihypertensive drugs (11, 22), amlodipine was chosen in this study because it is effective and safe for the treatment of hypertension in children and adolescents. In addition, as a long-acting drug, amlodipine is taken daily, which is convenient and helps improve patient compliance. In this study, we observed a BP control rate of 54.0% after one month treatment. In a previous study, Flynn et al. evaluated the amlodipine effect in 55 children with hypertension (89% had secondary hypertension) and found that amlodipine at the dosage of 0.16 ± 0.12 mg/kg/d for a treatment period of 6 ± 3 weeks effectively reduced BP (22). Subsequently, the long-term effect of amlodipine (≥ 6 months) was further investigated in 33 hypertensive children (6 with primary hypertension), which revealed that amlodipine was effective in BP reduction throughout the study period (20). Another multi-center study showed that the antihypertensive effect of amlodipine is dose-dependent (14). Conversely, we observed that amlodipine therapeutic efficacy is negatively associated with its dosages. This discrepancy, we speculate, may be due to the fact that all our patients had PH while only 31.3% of children in the other study had PH (14).

The Responders had a significantly higher baseline plasma ET-1 level than the Non-responders. Furthermore, ET-1 showed a significant contribution in the nomogram model. As a powerful vasoconstrictor, ET-1 may contribute to blood pressure elevation (15). In patients with moderate-to-severe hypertension, increased mRNA levels of prepro-ET-1, the precursor of ET-1, are found in the endothelium of small arteries (23). It has also been shown that higher plasma ET-1 concentrations are associated with higher risks of BP elevation and progression and incident hypertension (24, 25). However, the blood pressure regulation mechanism of ET-1 remains to be fully elucidated. Zeng et al. discovered that ET-1 activated the L-type calcium channel to increase intracellular calcium influx by stimulating NAD(P)H-derived superoxide production, suggesting an interaction between ET-1 and calcium channel activity (16). An animal study showed that CCB decreased ET-1 mRNA expression in the cardiovascular tissue of stroke-prone spontaneously hypertensive rats (26). Sudano et al. found that CCB attenuated ET-1-induced vasoconstriction in patients with essential hypertension (27). These data suggest that ET-1 contributes to hypertension by activating the calcium channel and CCBs may indirectly and directly inhibit ET-1 activity. Therefore, higher ET-1 levels may indicate a better response to CCBs.

In this study, the Non-responders were discovered to have a higher rate of hyperinsulinemia and IR. In the nomogram model, these two conditions were risk factors for ineffective probability. IR is involved in the etiopathogenesis of hypertension by activating the renin-angiotensin-aldosterone (RAS) system and inducing oxidative stress (28, 29). Accordingly, guidelines recommend that angiotensin converting enzyme inhibitors (ACEIs) should be the preferred antihypertensive agents for hypertensive patients with diabetes mellitus (8–10). Although amlodipine was reported to decrease oxidative stress biomarkers in patients with hypertension and type II diabetes, significant differences in HOMA index before and after treatment were not observed (30). Therefore, amlodipine may not be the first line choice in children with PH and IR.

Amlodipine is metabolized by cytochrome P450 oxidoreductase. A study of hypertensive patients following renal transplantation showed that the *CYP3A5* gene GG genotype was associated with a higher BP reduction than the GA and AA genotypes after amlodipine treatment (31). In the present study, however, we did not observe significant differences in the genotypes and allele frequencies of *CYP3A5* between Responders and Non-responders, in line with the finding described in other previous studies (32, 33).

### Study limitations

This study has several limitations. First, this is a single-center study with a small sample size. Second, the treatment period is short. Therefore, multi-center studies with large sample sizes to validate our findings are warranted.

## Conclusions

A nomogram model based on hyperinsulinemia, IR, ET-1 levels and amlodipine dosages for the prediction of amlodipine therapeutic efficacy in pediatric PH was established. This model showed good predictive performance and may be potentially applied to guide the selection of amlodipine for the treatment of pediatric PH.

## Data Availability

The original contributions presented in the study are included in the article/supplementary material. Further inquiries can be directed to the corresponding author.
